# Supplementary material for: Magnon bound states versus anyonic Majorana excitations in the Kitaev honeycomb magnet α-RuCl3
Source: Nat Commun. 2020 Mar 30;11:1603. doi: 10.1038/s41467-020-15370-1 (PMC7105467; doi:10.1038/s41467-020-15370-1)
Supplement: Supplementary file 1 — Supplementary Information [file 41467_2020_15370_MOESM1_ESM.pdf]

Supplementary Information

Magnon bound states versus anyonic Majorana excitations in the Kitaev honeycomb magnet α-RuCl$_3$

By Wulferding et al.

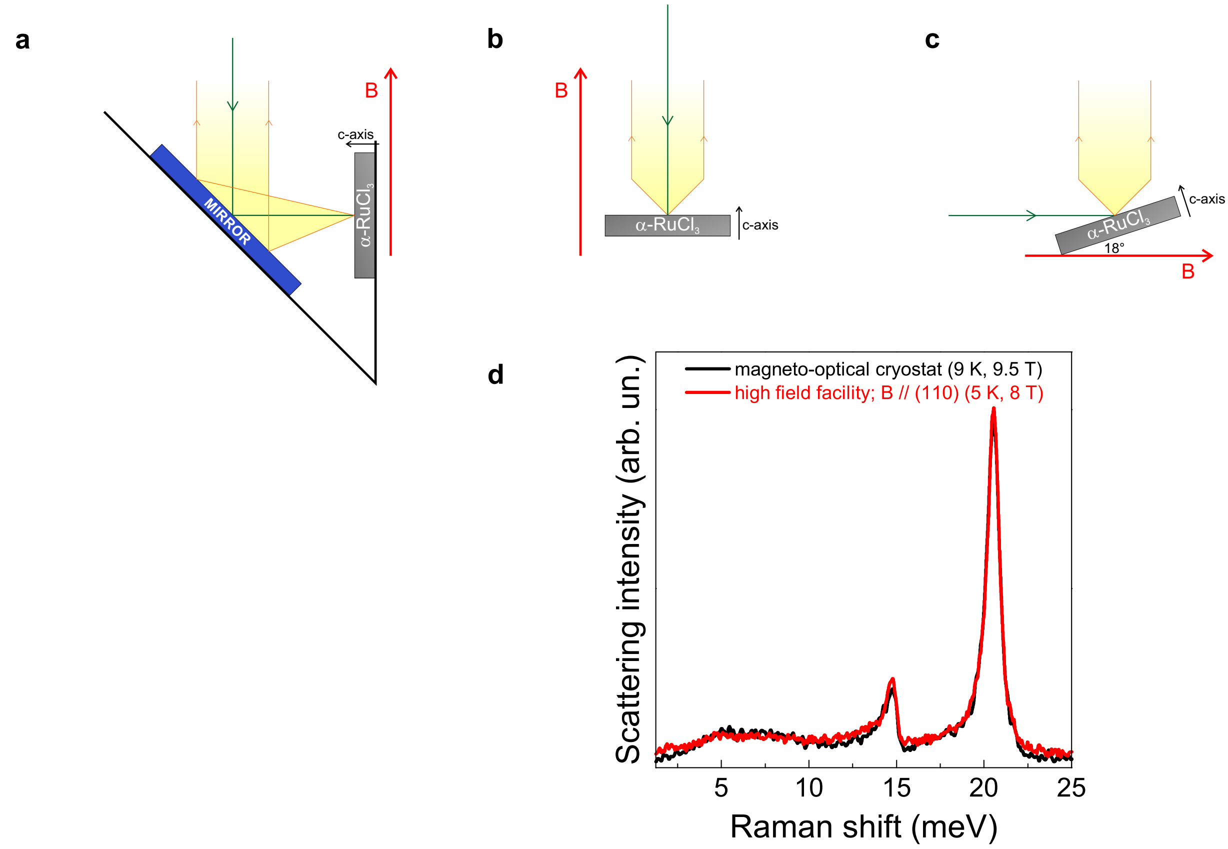

SUPPLEMENTARY FIGURE 1. Sketch of the scattering geometries and data comparison. **a**, Raman scattering in Voigt geometry. **b**, Raman scattering in Faraday geometry. **c**, 90° scattering geometry with the sample tilted by 18° from the in-plane field direction for the magneto-cryostat measurements. **d**, Comparison of Raman data obtained in the magneto-optical cryostat (black line; scattering geometry c) and in the high-field facility (red line, scattering geometry a).

Supplementary Note 1 | Scattering geometries

Raman scattering experiments with in-plane and out-of-plane magnetic fields have been carried out at the high magnetic field lab in Grenoble in Voigt- and in Faraday geometry, respectively, as sketched in Supplementary Figs. 1a and 1b. At present, the high-field Raman setup is not equipped with variable temperature inserts (VTIs). For a temperature scan, a magneto-optical cryostat with built-in VTI was used. These experiments were performed in 90° scattering geometry (Supplementary Fig. 1c) in applied magnetic fields up to 10 T. To achieve large enough in-plane field components, the sample was irradiated under a grazing angle of 18°. A maximum field of 10 T hence corresponds to an in-plane component of 9.5 T. In the main text and in the Supplementary all applied fields have been corrected

accordingly. All Raman experiments were performed using circular polarized light: incident right-circular polarized laser light (R) was created via a $\lambda/4$ waveplate, and backscattered light was passed through a left-circular polarized analyzer (L). This RL configuration allows us to probe the E_{g} symmetry channel, which carries the magnetic Raman contribution in the pure Kitaev model[1]. In Supplementary Fig. 1d we compare two spectra obtained from the magneto-optical setup and from the high-field facility. Despite the difference in temperature and scattering geometry, we find a nearly one-to-one correspondence.

Supplementary Note 2 | High field data

In the left panel of Supplementary Fig. 2 high-field Raman spectra taken at $T \geq T_\mathrm{N}$ are presented for magnetic fields applied along various crystallographic directions. In the right panel the corresponding color contour maps are given. While in-plane magnetic fields along the (100), (010), and (110) directions yield phenomenologically similar behavior concerning the intensity and spectral distribution of spectral weight, the energy scales of the emerging magnetic excitations are slightly different. This is owed to the finite in-plane anisotropy of α-$RuCl_3$.

A substantial spin-phonon-coupling is observed, as the $E_\mathrm{g}(1)$ mode gets heavily damped with increasing field through 10 T, while the $E_\mathrm{g}(2)$ mode is gradually suppressed. This is owed to the spectral redistribution of C: With increasing magnetic fields the continuum of fractionalized excitations is confined to higher energies as a gap opens and increases in size. In contrast, out-of-plane magnetic fields (along the 001-direction) do not affect the Raman data up to at least 29 T. This is consistent with the anisotropy of the extended Kitaev model.

Here we note that for magnetic fields along the (100) direction the phonon at 20 meV [$E_\mathrm{g}(2)$, corresponding to an in-plane displacement of Ru ions] appears to be splitting with increasing fields. To analyze this effect in detail we fit this spectral region with a sum of asymmetric Fano- and symmetric Voigt profiles (see Supplementary Fig. 3a-c). At low fields the excitation can be mainly described by a single Fano profile (green line), although a faint shoulder exists (blue line). With increasing fields the energies of these modes remain unaffected, but their intensity ratio changes, which results in a prominent growth of the shoulder feature. We assign the occurrence of these two lines to a lifting of the twofold degeneracy of the $E_\mathrm{g}(2)$ phonon, pointing towards a minute in-plane lattice distortion. As the energies of these two lines are unaffected by magnetic fields, this distortion is not a field-induced structural transition (see Supplementary Fig. 3d). With increasing field, a second, broader excitation approaches the 20 meV phonon energetically (dark red line), and around $B = 22$ T locks in with the phonon. This leads to a decrease in phonon lifetime, as evidenced by a decrease in intensity and a broadening in linewidth [see Supplementary Fig. 2 for B // (100)].

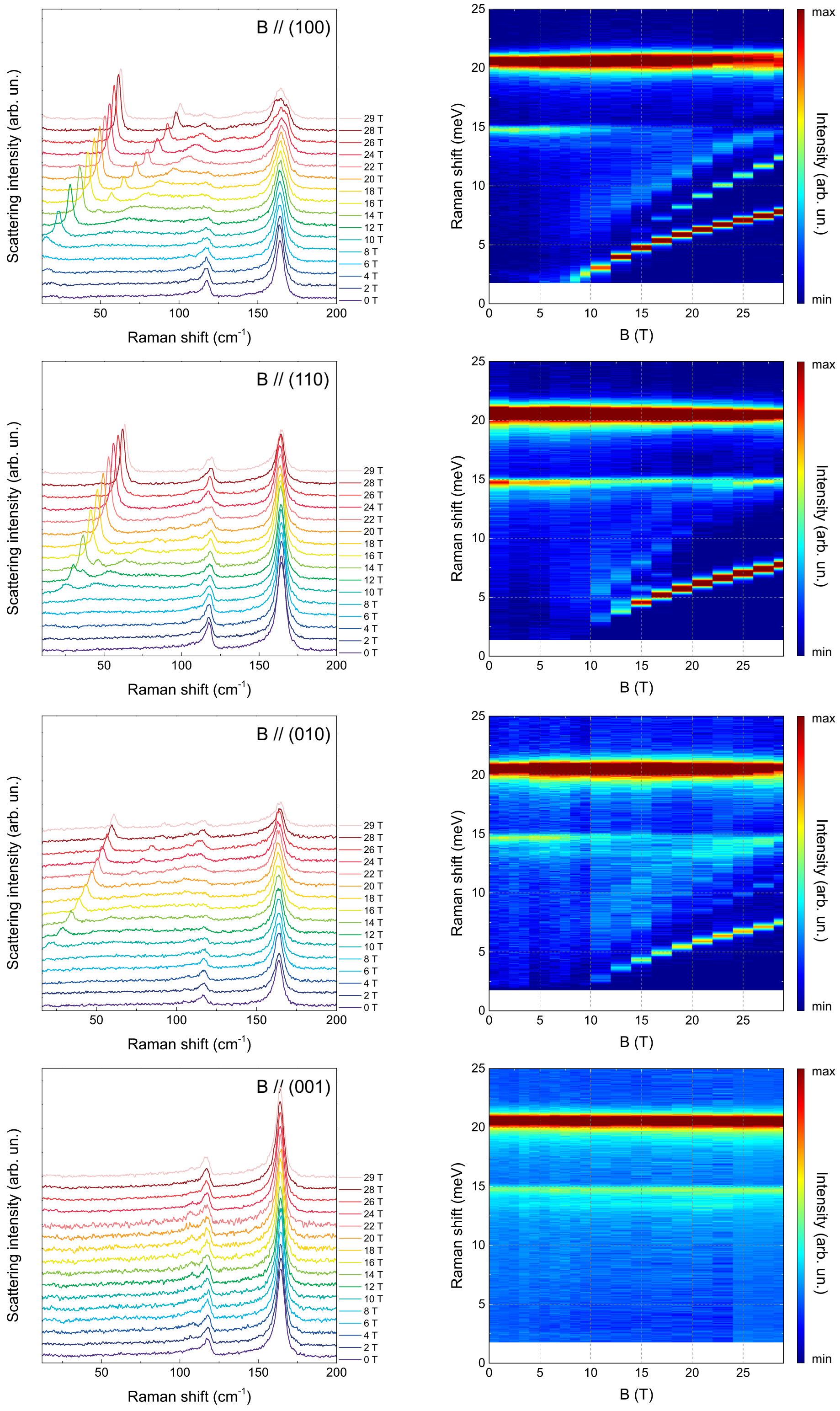

SUPPLEMENTARY FIGURE 2. **Raman data obtained over a wide field range in various field directions.** Left panel: As-measured Raman spectra for magnetic fields applied along the (100), (110), (010), and (001) directions. The spectra have been shifted vertically for clarity. Right panel: The respective color contour plots extending up to $E = 25$ meV.

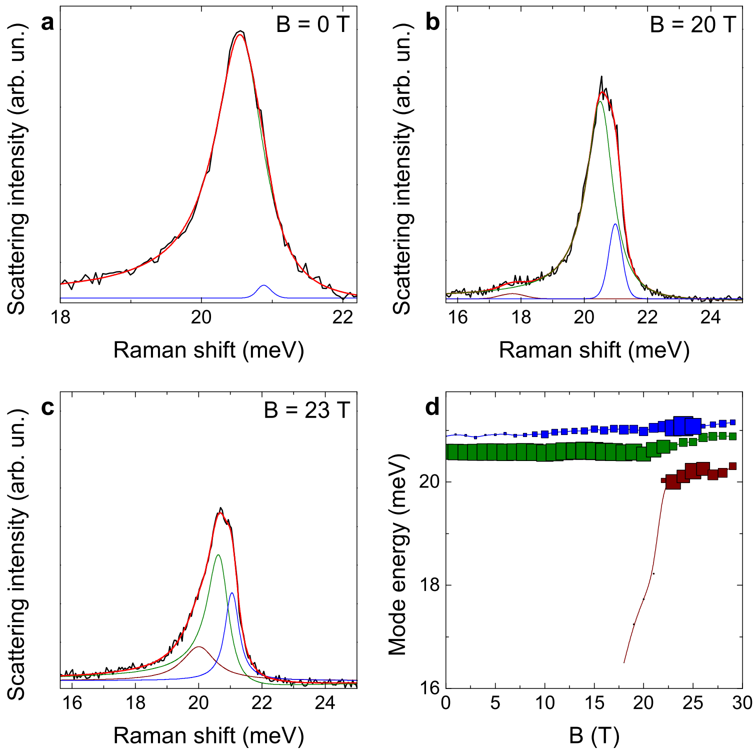

SUPPLEMENTARY FIGURE 3. **Field-induced phonon anomalies. a-c**, $E_{\mathrm{g}}(2)$ phonon at $T = 5$ K and magnetic fields along (100) direction of 0 T, 20 T, and 23 T, respectively. The black lines are as-measured data. The dark-red, green, and blue curves are fits to the individual excitations. The thick red curves are the sums of the individual fitting curves. **d**, Field-dependence of the individual modes energies. The symbol sizes are proportional to the modes' integrated intensities.

Supplementary Note 3 | Deconfined magnetic excitations at the critical field

In order to study the competition of quantum and thermal fluctuations and to analyze the statistics of excitation spectrum C in the intermediate field phase with suppressed long-range order we perform experiments as a function of temperature at a fixed magnetic field $B = B_{\mathrm{c}}$. This enables us to distinguish conventional bosonic multiparticle excitations from fractionalized Majorana excitations as quantum fluctuations deconfine the bosonic multiparticle excitations[2–4]. In Supplementary Figs. 4a and 4b we plot Raman spectra obtained at zero fields and around the quantum critical point at $B_{\mathrm{c}} = 6.7$ T, respectively, with temperatures ranging from 2 K – 290 K. The integrated intensity of C from 8.75 meV – 15 meV is shown in Supplementary Fig. 4c for $B = 0$ (blue diamonds) and $B = B_{\mathrm{c}}$ (red circles). This mid-energy regime of the continuum of Majorana fermionic excitations observed

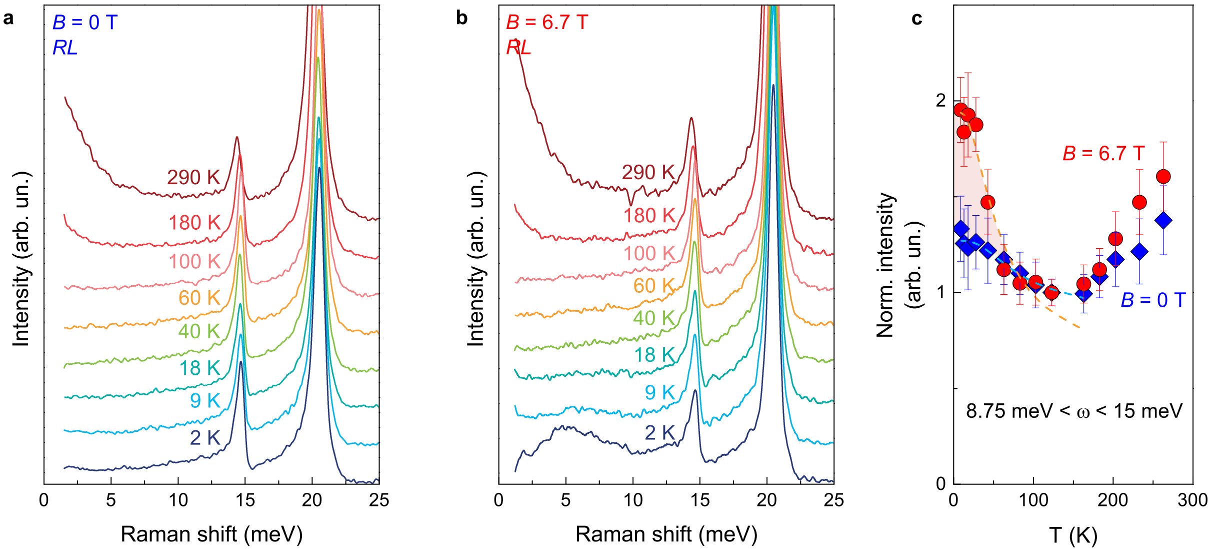

SUPPLEMENTARY FIGURE 4. Temperature dependence and analysis of the quantum statistics. a, Raman spectra obtained at $B = 0$ T in RL polarization over a wide temperature range 2 K – 290 K. **b**, Raman spectra obtained around $B_c = 6.7$ T in RL polarization over a wide temperature range 2 K – 290 K. **c**, Intensity of the broad continuum C integrated over a frequency range of 8.75 – 15 meV as a function of temperature at $B = 0$ T (blue diamonds) and $B = 6.7$ T (red circles). The dashed curves represent the two-fermionic contribution. Standard deviations are indicated by error bars.

in Raman spectroscopy arises from the simultaneous creation or annihilation of a pair of Majorana fermions. Its temperature dependence can be described by two-fermionic statistics, $I_{\rm MF} = [1 - f(\epsilon_1)][1 - f(\epsilon_2)]\delta(\omega - \epsilon_1 - \epsilon_2)$; with $f(\epsilon) = 1/[1 + e^{\epsilon/k_B T}]$ (see[4] for details). Additional terms that stem from deviations of the pure Kitaev model (Γ-term, Heisenberg exchange coupling) culminate in an additional bosonic background term, $I_{\rm B} = 1/[e^{\epsilon/k_B T} - 1]$. The thermal evolution of the continuum has been fitted to a superposition of both contributions. A more dramatic rise in intensity towards low temperatures is evident in the vicinity of quantum criticality. Similarly, the Fano asymmetry of the $E_g(1)$ phonon overlapping with C is more pronounced at B_c, indicative of a stronger coupling between lattice and Majorana fermionic excitations. Our observations suggest that the Majorana-related excitations at the quantum critical field B_c become more pronounced than the $B = 0$ T excitation, while developing the low-energy Majorana bound state MB. In Supplementary Figs. 5a and 5b we plot the as-measured continuum intensity for 0 T and 6.7 T, respectively, together with

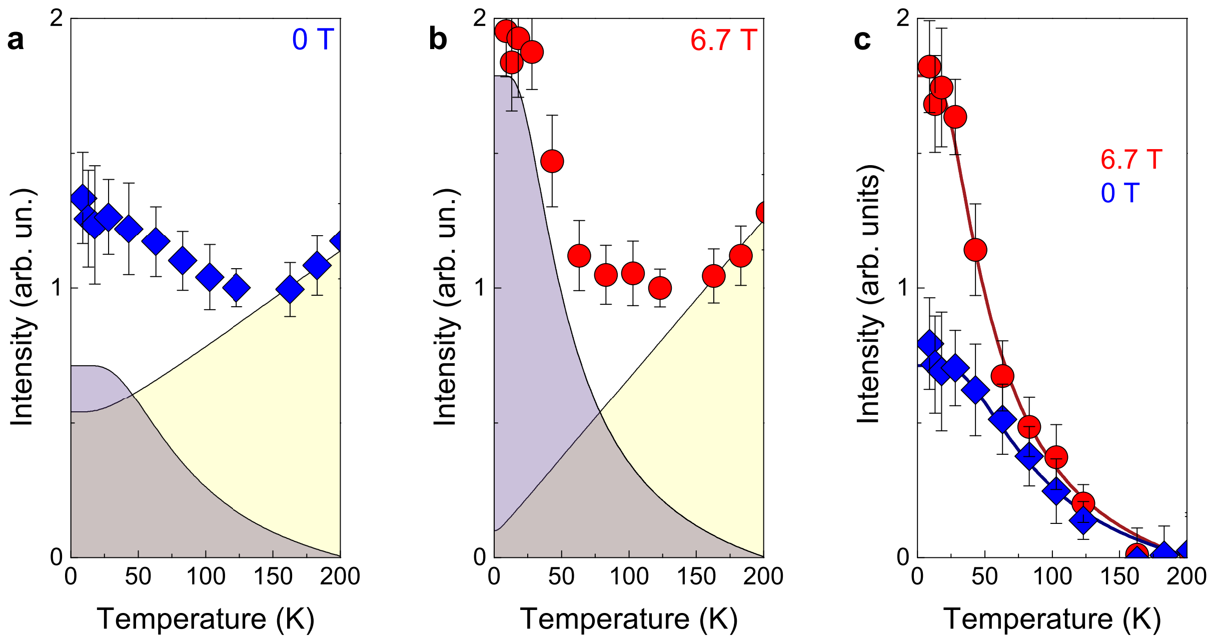

SUPPLEMENTARY FIGURE 5. Decomposition into bosonic- and fermionic excitations. a, Raman intensity of the mid-energy continuum at $B = 0$ T plotted as function of temperature. **b**, Raman intensity of the mid-energy continuum at $B = 6.7$ T plotted as function of temperature. The purple- and yellow-shaded backgrounds indicate two-fermionic and bosonic contributions, respectively. **c**, Raman intensity of the mid-energy continuum after subtracting the bosonic contribution. The two-fermionic contribution is marked by solid lines. Standard deviations are indicated by error bars.

their bosonic (yellow-shaded background) and two-fermionic (purple-shaded background) contributions. In Supplementary Fig. 5c we plot the continuum intensity after subtracting the bosonic contributions, with purely two-fermionic contributions remaining. In order to quantify the field-induced change in bosonic-to-fermionic excitations, we determine the ratio of integrated intensities of the two-fermionic ($I_{2-\mathrm{F}}$) and the bosonic (I_{B}) contributions in the temperature range 0 K – 200 K. We obtain $I_{2-\mathrm{F}} : I_{\mathrm{B}} = 0.40$ at $B = 0$ T, and $I_{2-\mathrm{F}} : I_{\mathrm{B}} = 0.89$ at $B = 6.7$ T. Thus, the two-fermionic contribution increased by about 120 % upon driving α-$RuCl_3$ towards quantum criticality, underlining that the field-induced phase is closer to a spin liquid than the zero-field phase.

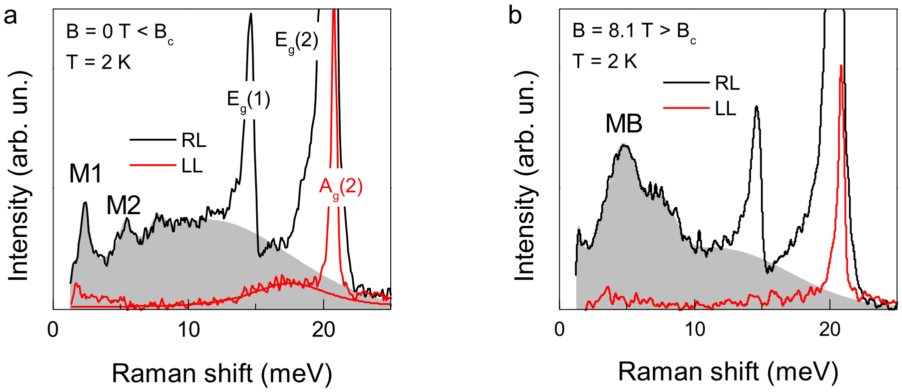

SUPPLEMENTARY FIGURE 6. Selection rule of magnetic excitations. a, b, Polarization-resolved Raman spectra at base temperature recorded at $B < B_c$ and $B > B_c$, respectively.

Supplementary Note 4 | Polarization dependence

Symmetry considerations can help identify the nature of observed emerging excitations. In Supplementary Figs. 6a-b we compare spectra measured at base temperature in RL (black line) and LL (red line) polarization, corresponding to the E_g and A_g symmetry channel, respectively. Notably, below as well as above B_c the spectral weight of magnetic excitations is clearly dominant in E_g symmetry and hardly discernible in LL polarization. Only a shallow weak background is detected with an onset energy of around 12 meV, suggesting that the continuum contains a minute contribution of A_g symmetry. In contrast, we do not find any signature of either *M1* or *M2* in A_g. Note that we also do not observe any leakage of E_g phonons in A_g scattering configuration and vice versa, signaling a very clean experimental polarization. The observed continuum coincides energetically with well-established reports of Majorana fermionic excitations[2,5]. Our very pronounced polarization dependence is in agreement with the theoretically predicted selection rules for the pure Kitaev model[1]. Therefore we can confidently assert that the broad scattering signal is dominated by excitations of Majorana fermions rather than magnonic multiparticles. The shallow, finite continuum in LL can be related instead to a deviation from the pure Kitaev model and arising from additional Heisenberg terms in the Hamiltonian. Above B_c, the continuum in LL is even more suppressed, suggesting that the magnetic field freezes out any detrimental impact of the weak perturbation terms. The fact that the intense, narrow mode MB at high fields obeys the same selection rules as the continuum, as well as the same selection rules as

the *M2* mode, gives evidence for a close relation between these excitations.

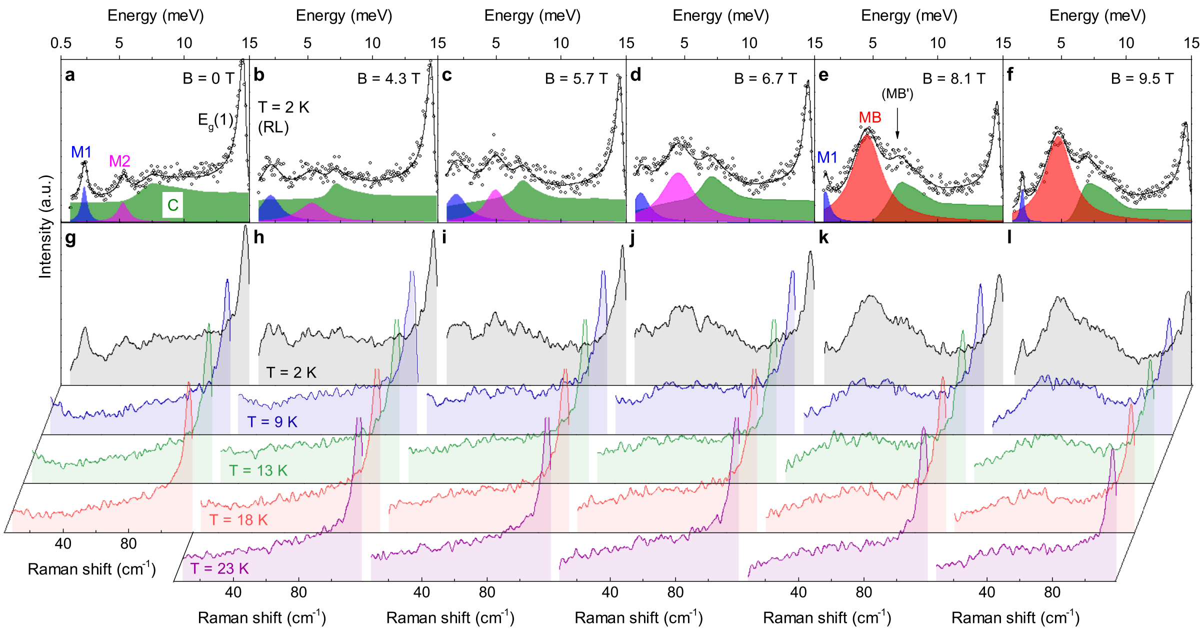

SUPPLEMENTARY FIGURE 7. **Field- and temperature dependence of Raman spectra. a-f**, Field dependence of Raman spectra at $T = 2$ K and in circular RL polarization. With increasing external fields through the critical regime $B_c \approx 6.7$ T, the gapless continuum (C; green shading) becomes gapped, while the higher-energy magnon mode (*M2*; purple) evolves into a bound state (*MB*). In contrast, the lower-energy magnon mode (*M1*; blue) persists across B_c. **g-l**, Thermal evolution of Raman spectra at the given applied magnetic fields.

Supplementary Note 5 | Temperature- and field dependence

In Supplementary Fig. 7 we present the full dataset of temperature- and field-dependent as-measured Raman data. The upper panel (a-f) presents the decomposition into various magnetic excitations at base temperature and with increasing magnetic field (see also main text). In Supplementary Figs. 7g-l the thermal evolution at a given field is shown. For $B < B_c = 6.7$ T, the magnon modes (*M1* and *M2*) rapidly disappear with increasing temperature above T_N. For $B > B_c$, the bound state (*MB*) is gradually suppressed with temperature, while a finite remnant spectral weight about 6.25 meV is observed even at $T = 23$ K. This implies that the nature of low-energy excitations is altered through B_c.

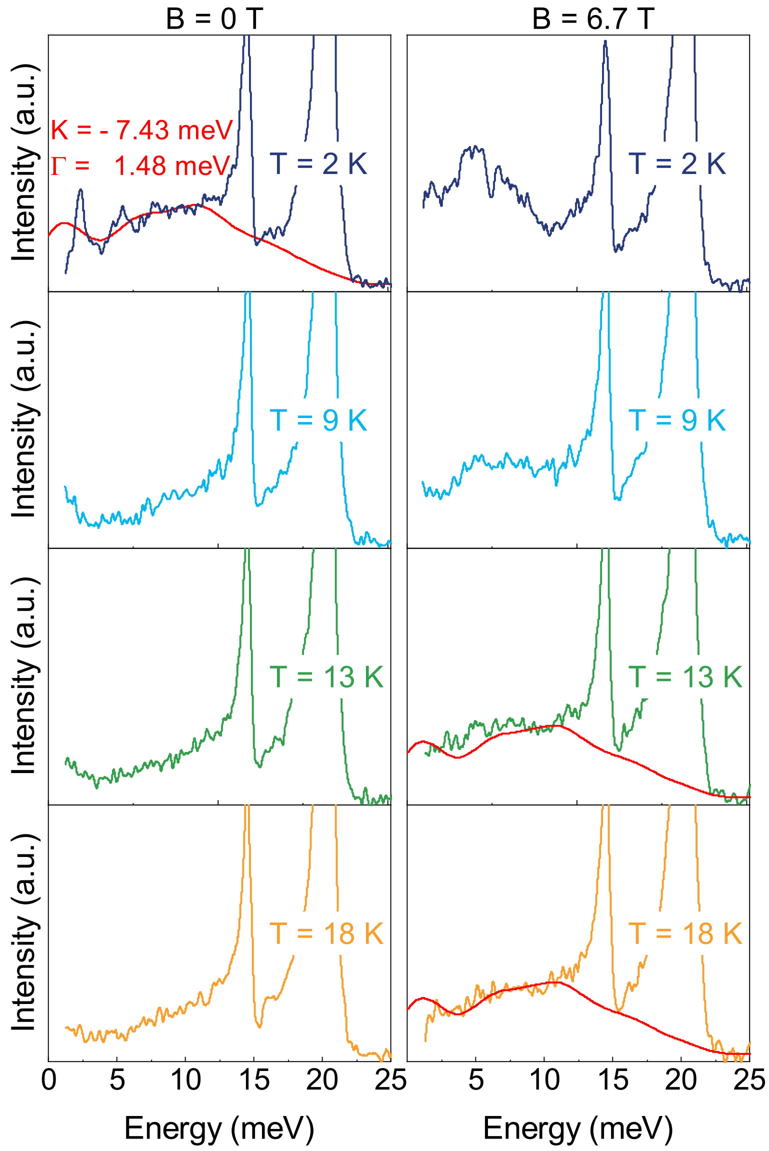

SUPPLEMENTARY FIGURE 8. $K - \Gamma$ **Model, Experiment vs. Theory.** As-measured temperature- and field-dependent Raman data together with calculations of the spectral weight based on the $K - \Gamma$ model[6] (solid red line). The parameters to fit the data were chosen as $K = -7.43$ meV and $\Gamma = 1.48$ meV, and the calculations were performed for small but finite temperatures.

Supplementary Note 6 | $K - \Gamma$ model: Theory vs. Experiment

Recent theoretical modeling of the Raman response in a Kitaev magnet has been extended to include a Γ-term. The key impact of the considerable Γ-term is to shift the spectral weight of itinerant Majorana fermionic excitations to a lower energy[6]. More specifically, the Γ-term on the one hand generates strong confinement (by opening a massive excitation gap), and

on the other hand, rearranges the spectral weight towards lower energies, offering abundant low-energy states. Consequently, the $K-\Gamma$ model is in favor of stabilizing low-energy bound states. At zero fields, the $K-\Gamma$ model gives a reasonable description of our $T = 2$ K data (see Supplementary Fig. 8) as the magnon modes contribute only small spectral weight. In contrast, at B_c, the itinerant Majorana fermions are confined to form the bound state at low temperatures. As a consequence, the $K-\Gamma$ model reproduces the Majorana continuum excitation at around the temperature where the Majorana fermions start to bind ($T = 13-18$ K). The comparison between our data and the $K-\Gamma$ model theory strongly supports the notion that the $K-\Gamma$ model can capture a key feature of the α-$RuCl_3$ magnetism possibly because the residual perturbations are weaker than the K and the Γ terms, and can be effectively quenched in applied magnetic fields and at slightly elevated temperature (see Supplementary Fig. 8).

Based on calculations of the dynamical spin response it was shown that with increasing strength of the anisotropy Γ-term a second mode can occur as a consequence of an inequality in gap size Δ_x and Δ_z[7]. This might account for the rather large linewidth of MB (see, e.g., Supplementary Fig. 7e). We can now estimate the binding energy E_B of the MB mode based on its energy $\omega_{MB} \approx 5$ meV and the gap size $\Delta \approx 6$ meV (see main text, Fig. 2h at $B = 9.5$ T) via $E_B = \Delta - \omega_{MB}$. This estimation yields a rough binding energy of 12 K. Considering that above 13 K the MB mode becomes ill-defined, melts and decays with the continuum (see Supplementary Fig. 9 and Fig. 3e, main text), our estimation proves to be a reasonable one. As the MB mode broadens in linewidth and gradually vanishes with increasing temperature, a narrow shoulder-like feature is observed around 5 meV (see $B = 9.5$ T at $T = 18$ K and 23 K). We tentatively assign this kink to a van-Hove singularity in the continuum. Note that such a structured continuum is well consistent with calculations for the $K-\Gamma$ model (see solid red lines in Supplementary Fig. 8).

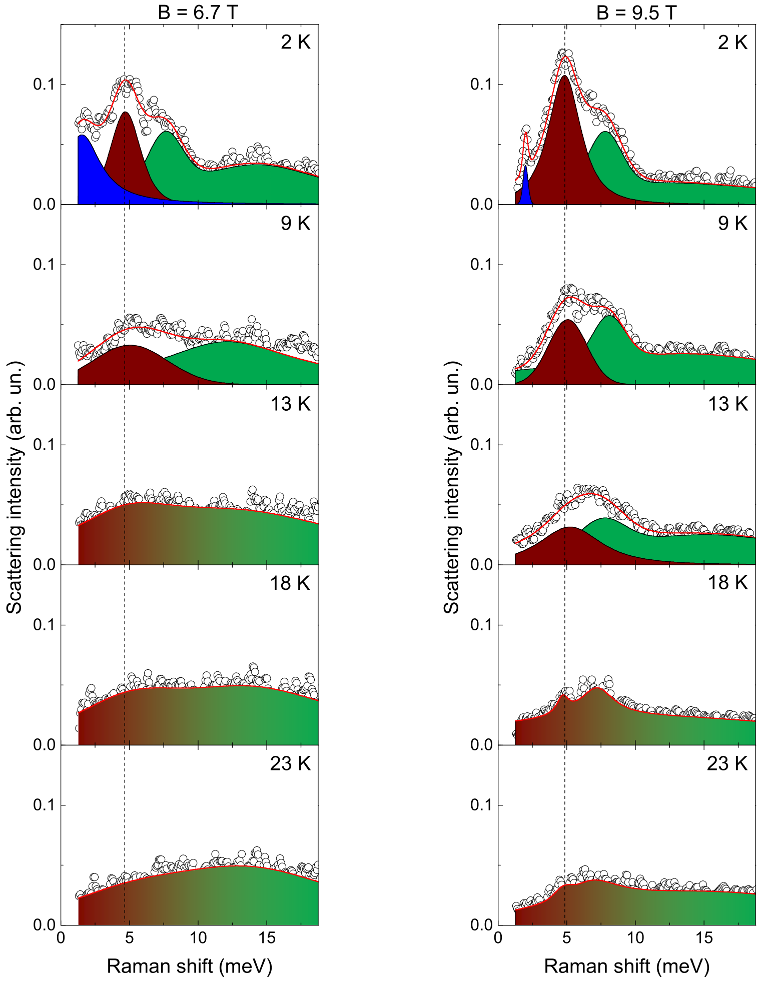

SUPPLEMENTARY FIGURE 9. **Thermal evolution of the *MB* mode at two different magnetic fields.** Phonon-subtracted spectra at B = 6.7 T and B = 9.5 T and at increasing temperature from 2 K to 23 K. Fits indicate the *M1* mode (blue), the *MB* mode (dark red) and the continuum of Majorana fermionic excitations (green).

Supplementary References

---

[1] Knolle, J., Chern, G.-W., Kovrizhin, D. L., Moessner, R. & Perkins, N. B. Raman Scattering Signatures of Kitaev Spin Liquids in A_2IrO_3 Iridates with A = Na or Li. *Phys. Rev. Lett.* **113**, 187201 (2014).

[2] Sandilands, L. J., Tian, Y., Plumb, K. W., Kim, Y.-J. & Burch, K. S. Scattering Continuum and Possible Fractionalized Excitations in α-$RuCl_3$. *Phys. Rev. Lett.* **114**, 147201 (2015).

[3] Glamazda, A., Lemmens, P., Do, S.-H., Choi, Y. S. & Choi, K.-Y. Raman spectroscopic signature of fractionalized excitations in the harmonic-honeycomb iridates β- and γ-Li_2IrO_3. *Nat. Commun.* **7**, 12286 (2016).

[4] Nasu, J., Knolle, J., Kovrizhin, D. L., Motome, Y. & Moessner, R. Fermionic response from fractionalization in an insulating two-dimensional magnet. *Nat. Phys.* **12**, 912 (2016).

[5] Glamazda, A., Lemmens, P., Do, S.-H., Kwon, Y. S. & Choi, K.-Y. Relation between Kitaev magnetism and structure in α-$RuCl_3$. *Phys. Rev. B* **95**, 174429 (2017).

[6] Rousochatzakis, I., Kourtis, S., Knolle, J., Moessner, R. & Perkins, N. B. Quantum spin liquid at finite temperature: Proximate dynamics and persistent typicality. *Phys. Rev. B* **100**, 045117 (2019).

[7] Knolle, J., Kovrizhin, D. L., Chalker, J. T. & Moessner, R. Dynamics of fractionalization in quantum spin liquids. *Phys. Rev. B* **92**, 115127 (2015).
